# Biodegradation of Navy N5RL1 carpet dye by *Staphylococcus saprophyticus* strain BHUSS X3

**DOI:** 10.1007/s13205-015-0276-7

**Published:** 2015-01-31

**Authors:** Lata Kumari, Ajay Kumar Verma, Dhanesh Tiwary, Deen Dayal Giri, Gopal Nath, Pradeep Kumar Mishra

**Affiliations:** 1 Department of Chemistry, Indian Institute of Technology (Banaras Hindu University), Varanasi, India; 2 Department of Chemical Engineering and Technology, Indian Institute of Technology (Banaras Hindu University), Varanasi, India; 3 Department of Microbiology, Institute of Medical Sciences (Banaras Hindu University), Varanasi, India

**Keywords:** Biodegradation, *Staphylococcus saprophytic* BHUSS X3, Carpet industry, Navy N5RL1

## Abstract

Biodegradation of Navy N5RL1, a widely used acidic azo dye in carpet industry, was studied by bacterial strain isolated from the dye-contaminated soil collected from a carpet industry premises located in Bhadohi, Sant Ravidas Nagar and Uttar Pradesh, India. The isolated strain was identified as *Staphylococcus saprophyticus* BHUSS X3 on the basis of morphological, biochemical and 16S rRNA gene sequencing analysis. The strain BHUSS X3 decolorized 95.7 % of dye (100 mg/l) within 6 h at optimum pH 8, temperature 35 °C, inoculum 4.0 % under static condition during 24 h incubation. The isolated bacterial strain BHUSS X3 can toralate dye concentration upto 1,000 mg/l. The dye degradation metabolites were confirmed by analysis of degraded products using UV–Vis spectrophotometric, HPLC and FTIR technique. The phytotoxicity analysis was also conducted on *Phaseolus aureus* and enhanced seed germination was recorded.

## Introduction

The synthetic azo dyes are widely used in carpet, textile, paper, cosmetics, pharmaceuticals, food and many other industries (Saratale et al. 2011). Their annual production exceeds 7 × 10^7^ metric tons (Robinson et al. 2001; Akhtar et al. 2005) and demand is continuously increasing. About 70 % of azo dyes produced are applied in textile industry due to their availability in variety of colors, high stability and low cost (Wang et al. 2008; Saratale et al. 2009). A significant proportion of total production (~10–15 %) remains unutilized and released in the environment at various stages that contaminate water bodies (like river, lake, ground water) (Ozdemir et al. 2013; Moosvi et al. 2007; Prasad and Rao 2013). The recalcitrant azo dye effluents adversely affect living organisms and their ecosystem. They are mutagenic and carcinogenic for human. The intense color reduces photosynthetic activity of aquatic plants leading to low dissolved oxygen level and anoxic conditions, causing killing of aquatic organism, including fishes. The effluent discharged from several types of industries has proven mutagenic activity (Coelho et al. 1992). It is therefore necessary to treat such industrial wastewater.

There are variety of physico-chemical methods such as flocculation/coagulation (Rodrigues et al. 2013) precipitation, sedimentation, reverse osmosis and nanofiltration (Myung et al. 2005). However, these methods are associated with low dye removal efficiency and high cost due to high energy requirement (Forgacs et al. 2004; Zhang et al. 2004). In contrast, bioremediation of dye effluents by microorganisms is cost effective and ecofriendly (Kuhad et al. 2004; Mohana et al. 2007). Recently, researchers have focused on isolating microorganisms from contaminated sites to degrade dye effluents under aerobic and anaerobic conditions. Several microorganisms such as *Morganella* sp. HK-1, *Bacillus cereus* strain HJ-1 and *Yarrowia lipolytica* have been identified for efficient degradation of aromatic dyes (Pathak et al. 2011).

During the anaerobic degradation, harmful aromatic amine intermediates are produced, which are carcinogenic and mutagenic. These harmful intermediates are converted to less toxic products by hydroxylation and deamination reactions using aerobic biodegradation steps (Tripathi and Srivastava 2011; Levine 1991). As there is draft of literature regarding the degradation and detoxification of Navy N5RL1 dye, being used for coloring the carpet, present investigation aims to investigate its degradation using bacterial strain (*Staphylococcus saprophyticus* BHUSS X3) isolated from dye-contaminated site. The various factors affecting dye degradation such as agitation speed, pH, initial concentration and temperature were optimized. The decolorization and degradation products were analysed by UV–Vis Spectrophotometer, FTIR and HPLC. Further, phytotoxicity of degradation products of Navy N5RL1 dye was tested by germinating the *Phaseolus* seeds on these degradation products.

## Materials and methods

### Chemicals and nutrient media

The dye Navy N5RL1 was procured from Amit Carpet Industries Limited, Sant Ravidas Nagar (Bhadohi), Uttar Pradesh, India. All the solvents used in the HPLC were of HPLC grade (Fisher Scientific). The nutrient media used for culturing the bacteria were purchased from Hi-Media, India.

### Isolation and identification of bacteria

Dye-contaminated soil samples were collected from Sant Ravidas Nagar (Bhadohi), Uttar Pradesh, India. Ten milliliters of aqueous suspension of 1 g of soil was mixed in 100 ml nutrient broth containing 5 g peptone, 5 g NaCl, 1.5 g yeast extract and 1.5 g beef extract per litter for isolation of bacteria. The nutrient was supplemented with 500 mg/l Navy N5RL1 dye and incubated at 30 ± 2 °C in an orbital shaker (100 rpm) as well as under static condition for 48 h to enrich the dye-degrading bacterial population. One milliliter of the culture was withdrawn after 2 days of incubation and it was serially diluted. 0.1 ml samples were withdrawn from 10^−4^ dilution and these were incubated on nutrient agar plate containing 500 mg/l Navy N5RL1 dye. These plates were incubated till appearance of morphologically distinct bacterial colonies. The selected colonies were further streaked on fresh nutrient agar plates for isolating the pure strains. Selected fast-growing bacteria were again streaked over the fresh nutrient agar plate. For further utilization, these bacterial colonies were stored in nutrient agar slant at 4 °C and glycerol stock at −20 °C. The bacterial strains were identified morphologically, biochemically as well as through 16S rRNA gene sequence analysis. For 16S rRNA gene sequence analysis, overnight grown bacterial cultures in nutrient broth were centrifuged at 10,000 rpm for 10 min. The pellets were washed with 1 % Triton-X buffer and treated with proteinase K for digestion (5 µl in 1 % SDS, 55 °C for 1 h). The bacterial DNA isolation was done by CTAB method. For this, bacterial cell pellets were mixed with 1 % CTAB and 0.7 M NaCl and incubated in water bath at 65 °C for 10 min. The mixture was cooled and mixed with phenol:chloroform:IAA (25:24:1) in vortex for 15 s and then centrifuged at 10,000 rpm for 10 min. The supernatant thus collected was mixed with equal volume of chloroform:IAA (24:1). One milliliter of isopropanol was added to it and resultant solution was kept for 10 min at room temperature (25 °C). The pellets thus obtained were washed with 70 % ethanol, dissolved in TE buffer and stored at −20 °C for amplification of DNA using PCR. PCR was performed using Taq DNA polymerase and 16S universal primers (NF5′GGCGGCAKGCCTAAYACATGCAAGT3′ and NR5′GACGACAGCCATGCASACCTGT3′). The PCR amplified products were purified by utilizing the purification kit (Hi-Media) prior to DNA sequencing. The resultant 16S rRNA sequence was analysed using BLAST of NCBI for identifying closest relative of the bacteria and the sequence was then deposited in Gene Bank (Accession number KJ439577). The 16S rRNA gene sequence of bacterial strain BHUSS X3 was aligned with related species of the genus *Staphylococcus*, *Bacillus* and *Pseudomonas* using MEGA 6.

### Inoculum preparation

The cultivated loopful bacterial culture was inoculated in 100 ml of nutrient broth and incubated (100 rpm, 30 °C, 24 h). The inoculum size of 0.1 ml of actively growing culture (~10^7^ cells/ml) was selected for the optimization of various parameters for dye degradation experiments. Another broth under identical conditions was used as control without bacterial culture.

### Decolorization studies

One milliliter of inoculum was incubated in 100 ml nutrient broth for 24 h and then 1 ml of dye stock containing 10,000 mg/l dye was added to it. Two-milliliter samples were withdrawn at regular interval, centrifuged (10,000 *g* for 15 min), and supernatant thus obtained was analyzed for estimation of degradation efficiency using double beam UV–Vis spectrophotometer in 200–700 nm range (Systronics 2202). The percentage decolorization was calculated as given below:

$$ {\text{Decolorization (}}\% )= \frac{{{\text{initial}}\, {\text{OD}} - {\text{final }}\,{\text{OD}}}}{{{\text{intial}}\,{\text{OD}} }} \times 100. $$

### Optimization of dye degradation parameters

The optimization studies were carried in pH range 4–10, temperature range 20–45 °C, initial dye concentration (100–1,000 ppm) at static condition as well by shaking at 100 rpm for degradation of Navy N5RL1 dye. Each experiment was performed in triplicate.

### Analytical investigation

#### HPLC and FTIR analysis

The HPLC and FTIR were performed for estimation of degradation efficiency and degradation products. The degraded samples (100 ml) were centrifuged at 10,000 rpm for 15 min at 25 ± 1 °C, and mixed with equal volumes of ethyl acetate and kept in the desiccators for water removal using anhydrous sodium sulphate. The solution was further dried using rotary evaporator. The control was also exposed to similar step. The residue thus obtained was dissolved in 5 ml of methanol and subjected to HPLC analysis (Water HPLC, Model no. 2690) on a reverse phase C18 column (5 mm, 4.6, 250 mm) at 35 °C. Acetonitrile at 10–90 % gradient was used as the mobile phase and the flow rate was adjusted to 1.0 ml/min. Analysis was carried out for 20 min and the peaks were identified using a photodiode array detector at 294 nm.

The FTIR spectra of pure and degraded dye samples were analysed between wave number 4,000 and 500 cm^−1^ by mixing them in KBr pellet using FTIR spectrophotometer (Perkin Elmer, version 10.03.05).

#### Phytotoxicity study

The phototoxicity of Navy N5RL1 dye was assessed before and after bacterial degradation of the dye in the concentration range of 100 and 1,000 mg/l using *Phaseolus aureus* seeds. A piece of sterilized filter paper was soaked in selected dye concentration prior and after the degradation with bacterial strain. The surface-sterilized seeds were placed over it and incubated in the seed germination chamber. Filter papers were moistened with the same amount of degraded and undegraded dye solution intermittently. The control was run by keeping the paper pad wet with tap water. Length of plumule and radical was measured after 6 days and percentage of germination was calculated.

## Results and discussion

### Bacterial identification by 16S rRNA gene sequencing

**Fig. 1:**
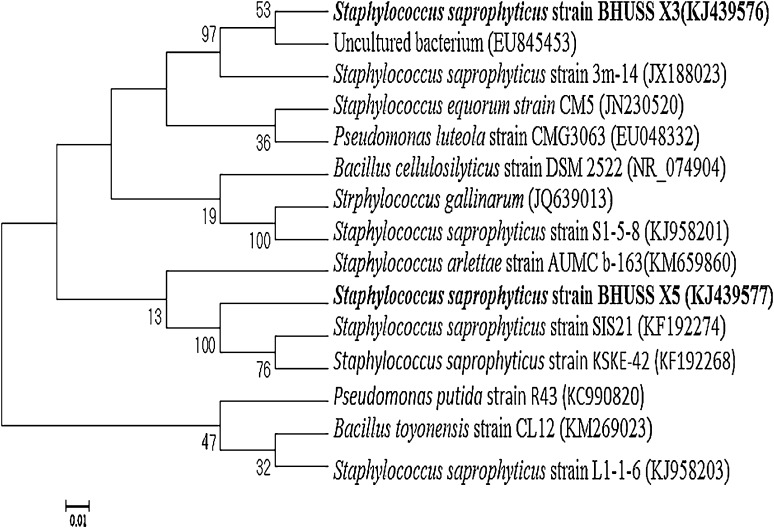
The evolutionary history of bacterial strain BHUSS X3 was analysed using the neighbor-joining method, bootstrap values (*n* = 1,000) are indicated at the nodes and scale bars represent 0.01 substitution/site; the sequences have been retrieved from NCBI database, showing the phylogenetic relationships of *Staphylococcus saprophytic* BHUSS X3 with different strain of *Staphylococcus* and other species of genus *Pseudomonas* and *Bacillus*

The fast-growing Gram-negative bacteria on the dye-amended nutrient agar was initially indentified morphologically and biochemically (Table 1) before their molecular identification using 16S rRNA gene sequence technique. The bacterial 16S rRNA sequence was most closely related to *Staphylococcus* *saprophyticus* BHUSS X3. The evolutionary relations of the strain among the genus of *Staphylococcus* are shown in the phylogenetic tree (Fig. 1). The sequence of the strain was directly deposited in Gene Bank (Accession No. KJ439577) (http://www.ncbi.nlm.nih.gov/nuccore/KJ439577). The analysis of 1,150 base pair of 16S rRNA gene sequence obtained was 99 % identical to that of *Staphylococcus* *saprophyticus* compared to the other genera.

**Table 1:** Characteistics of isolated bacterial strain *Staphylococcus saprophytic* BHUSS X3

Characteristic	*Staphylococcus saprophytic* BHUSS X3
Gram staining	−ve
Shape	Cluster, chain
SIM motality	Non-motile
TSI	+ve/no growth
H_2_S production	+ve
Glucose utilization	+ve
Sucrose utilization	+ve
Lactose utilization	+ve
Citrate utilization	−ve
Urease	−ve
Oxidase	−ve
Indol	+ve

### Parameter optimization for degradation studies

#### Effect of agitation

**Fig. 2:**
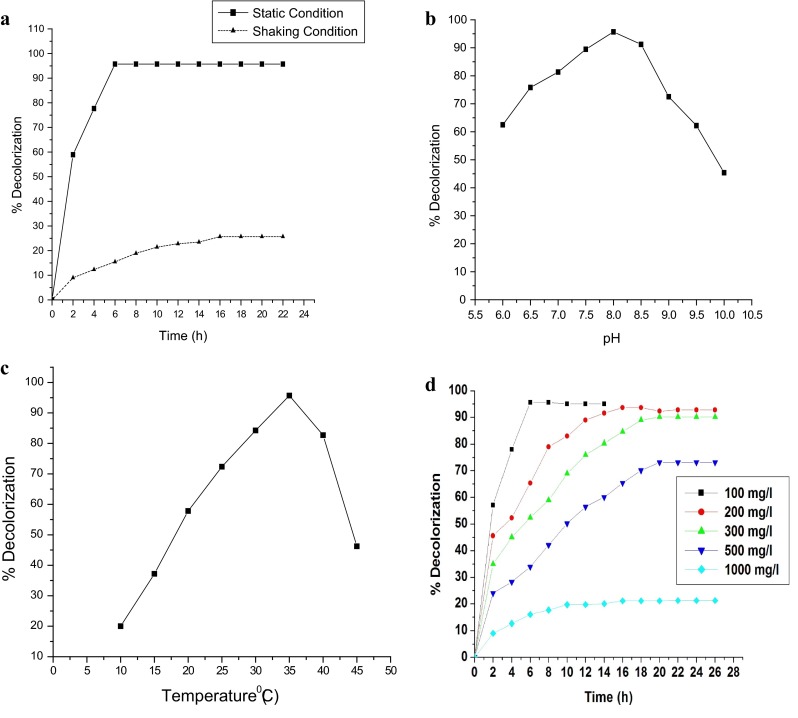
**a** Effect of static/shaking condition for efficient decolorization of dye Navy N5RL1 by *Staphylococcus saprophytic* BHUSS X3. **b** Effect of pH on dye Navy N5RL1 decolorization by *Staphylococcus saprophytic* BHUSS X3 optimized static culture condition at pH 8.0, 35 °C. **c** Effect of temperature for decolorization of dye Navy N5RL1 by *Staphylococcus saprophytic* BHUSS X3. **d** Effect of initial dye Navy N5RL1 concentrations (100–1,000 mg/l) under optimized static culture condition at pH 8.0, 30 °C

The bacteria decolorized Navy N5RL1 dye more efficiently under static anoxic conditions (95.7 %) as compared to the agitation at 100 rpm (25 %) for initial dye concentration of 100 mg/l, for 6 h incubation as shown in (Fig. 2a). The bacterial growth was fast under shaking condition but colour removal was slow. In contrast, under static condition, bacterial growth was slow but dye decolourization was comparatively fast. The result could be explained in terms of facultative anaerobic nature of isolated bacteria *Staphylococcus saprophyticus* strain BHUSS X3. Under static condition, partial anaerobic or microaerobic condition is generated and bacteria excrete extracellular and intracellular enzymes for dye decolourization. In the agitated samples of the dye, the bacterial respiration consumes most of the NADH necessary for the azoreductase activity in the dye decolourization (Stolz 2001). Therefore, static conditions were adopted to investigate dye decolorization in the following experiments.

#### Effect of pH

The bacteria *Staphylococcus saprophyticus* BHUSS X3 was able to tolerate broad pH range of 6–10, so decolourization was tested for this range. For initial dye concentration, 100 mg/l, degradation increased with increasing pH up to 8 (95.5 %) and further increase in pH reduced the decolorization efficiency (Fig. 2b). The decolorization was affected by the extreme pH. The dye degradation under alkaline conditions has been reported to be favoured by various bacteria (Chen et al. 2003; Guo et al. 2007; Kilic et al. 2007; Bhatt et al. 2005) probably due to preferred growth in basic pH.

#### Effect of temperature

The incubation temperature is an important parameter for bacterial growth. The selected mesophilic bacteria were utilized for dye degradation in temperature range 20–45 °C as shown in Fig. 2c. It is clear from the figure that percentage decolorization increased with increase in temperature up to 35 °C and further increase in temperature beyond 35 °C resulted in decreased dye degradation. The result could be explained in terms of the inactivation of enzyme and loss of cell viability (Saratale et al. 2009).

#### Effect of initial dye concentration

The dye degradation experiment was conducted for the initial concentration range of 100–1,000 mg/dye. The dye decolourized most **e**ffectively in initial concentration of 100 mg/l dye (95.7 %) within 6 h (Fig. 2d). The decolorization decreased as the initial concentration of dye increased. In case of 500 mg/l initial dye concentration degradation declined to 65 % in 18 h. Such reduction in the decolourization rate at higher concentrations has been attributed to toxic effect of dye (Jadhav et al. 2011; Kalme et al. 2007; Khera et al. 2005).

### Analytical investigation

#### UV–Vis spectral analysis

**Fig. 3:**
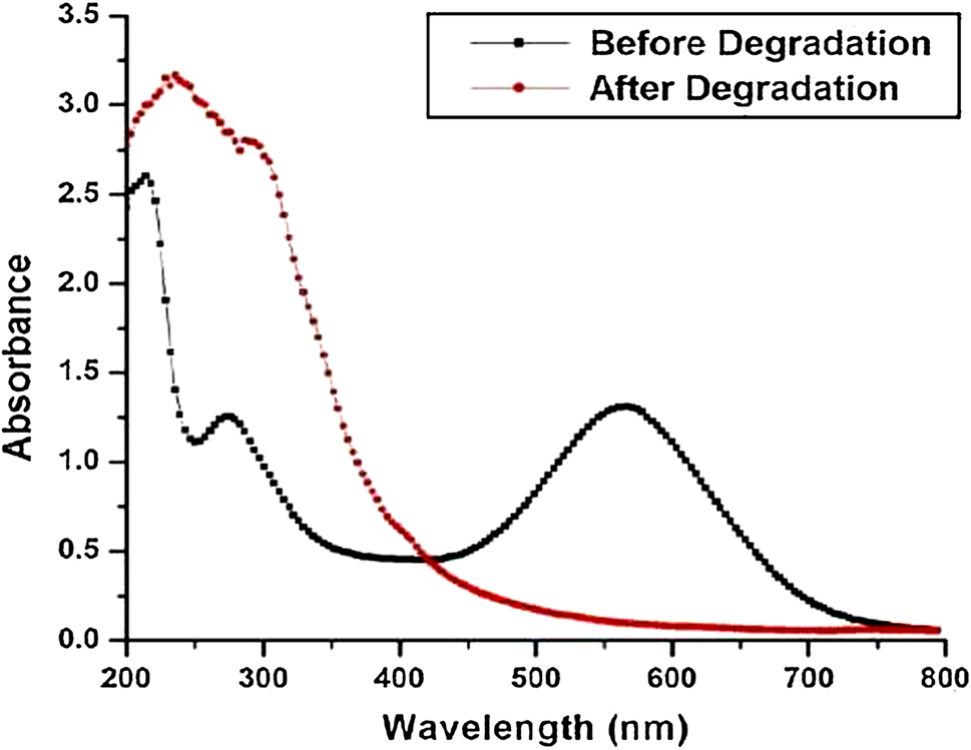
UV–Vis spectrophotometer of Navy N5RLI (100 ppm) biodegraded by *Staphylococcus saprophytic* BHUSSX3 before and after optimized condition at *T* = 35 °C, pH 8.0, bacterium = 2.6 × 10^6^ cells/ml

In the UV–Vis spectral analysis of pure dye solution, there were two minor peaks at 210, 260 nm and a major peak at 560 nm as shown in Fig. 3. The peak in the visible range disappeared after decolorization of the azo dye by bacteria and two peaks in the UV range were replaced by single peak at 250 nm (Fig. 3) probably due to the formation of some aromatic intermediate products.

#### HPLC analysis

**Fig. 4:**
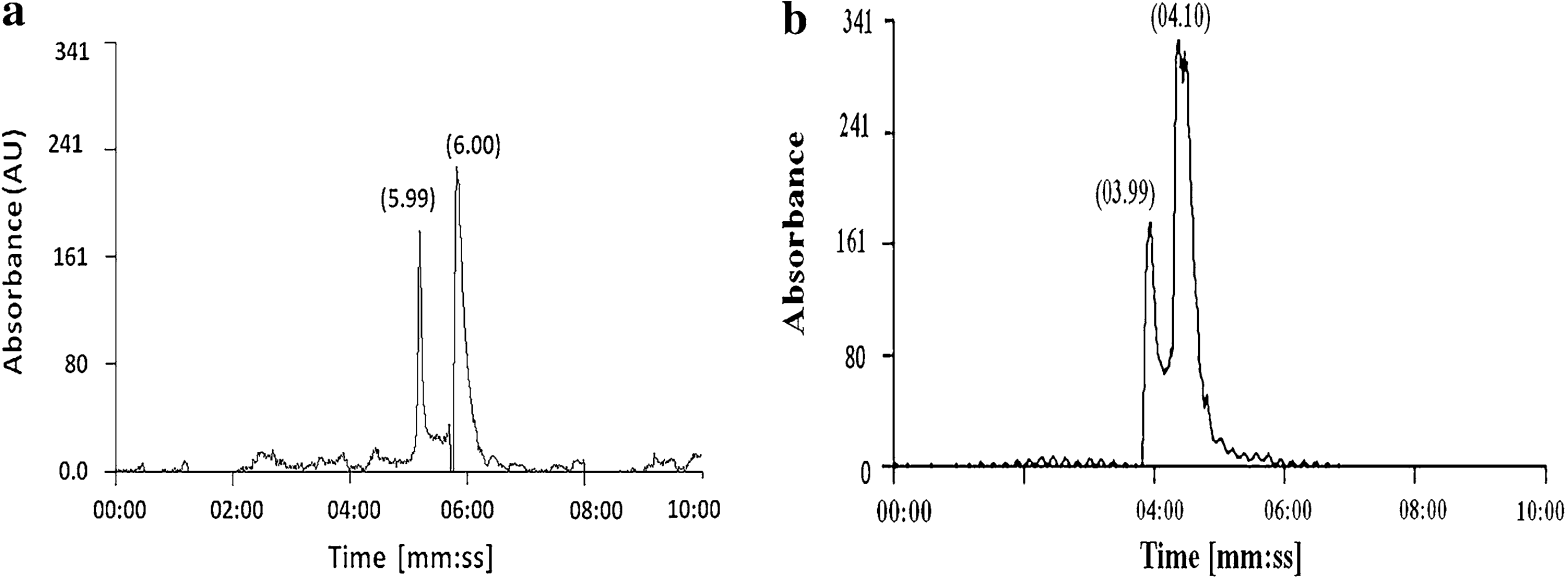
HPLC analysis for dye degradation, **a** dye control sample, **b** degraded sample

The HPLC analysis of dye before degradation has two peaks at the retention time 5.99 and 6.00 min, whereas degradation products showed (Fig. 4a, b) peaks at lower retention time namely 3.99 and 4.10 min, probably due to degradation of dye into small intermediate products.

#### FTIR analysis

**Fig. 5:**
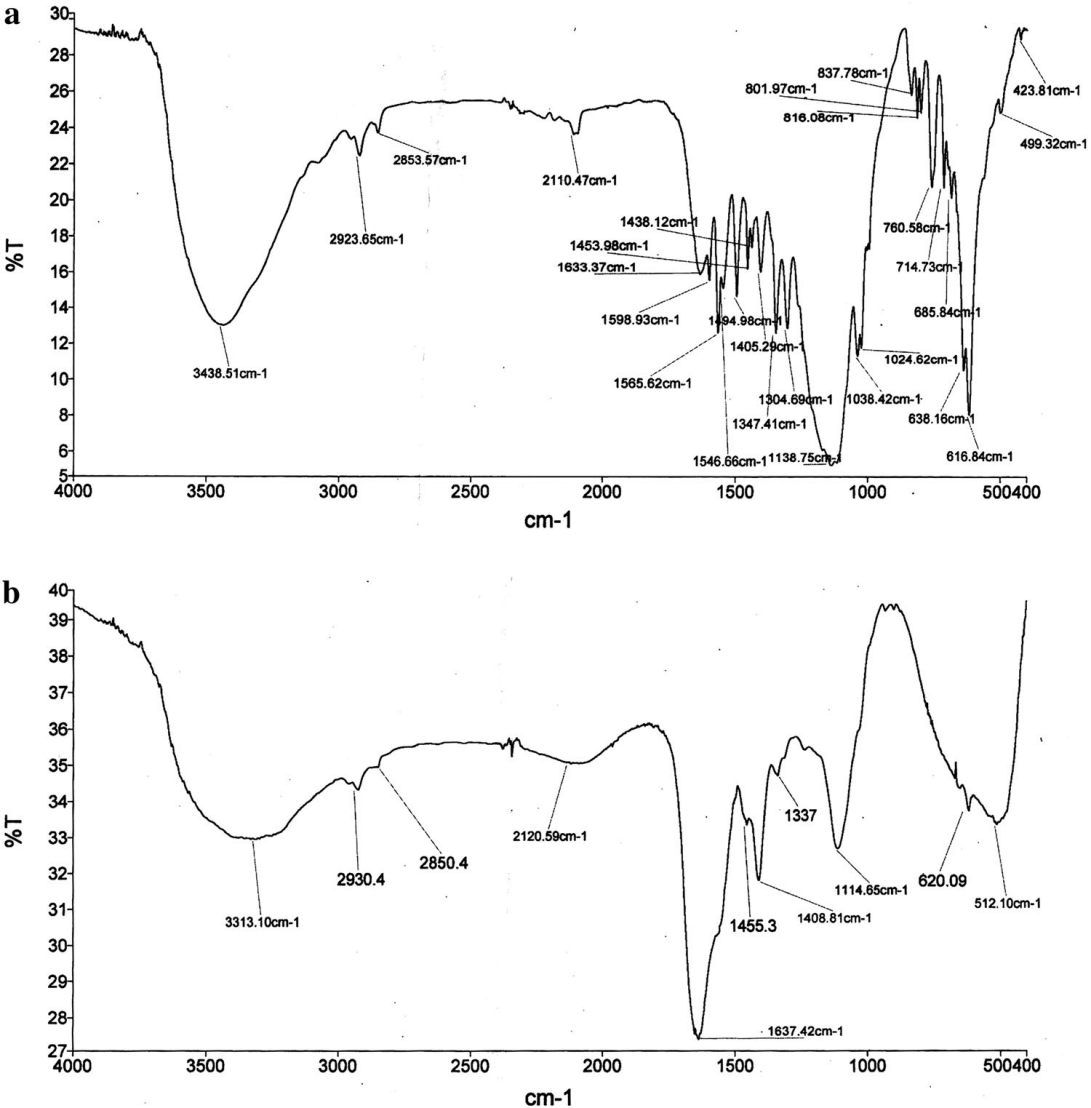
FTIR analysis of Navy N5RL, **a** before degradation, **b** after degradation

The FTIR spectra of dye of (Fig. 5a) and dye degradation products differed with number of peaks and their positions (Fig. 5b). The appearance of peak at 1,598.93 cm^−1^ confirmed the presence of aromatic nitro compound and azo group in dye whereas peaks at 1,494.98 and 1,565.62 cm^−1^ were related to aromatic nitro compounds. The C–C stretching is indicated by peaks at 2,923.65 and 2,853.57 cm^−1^. The peaks at 1,347.41 and 1,138.75 cm^−1^ are due to the presence of the sulphonated dye compound. The peak at 1,038.81 cm^−1^ indicates primary alcoholic group and peak at 837 cm^−1^ indicates C–H deformation of benzene ring. The sharp peak at 1,598.93 cm^−1^ in azo compounds absent in the FTIR spectral analysis of degraded products confirms the cleavage of azo bonds. The peak at 3,313.10 cm^−1^ due to O–H stretching indicates hydroxylation of the product. A significant change in FTIR spectrum in degraded dye sample which displayed peaks at 3,230 and 1,667 cm^−1^ for–OH stretching and a peak at for C=N respectively. Fermi resonance band at 2,930 cm^−1^ for –CH_3_ supported by a peak at 1,455 cm^−1^ for –CH_3_ asymmetric bending vibration indicates the formation of oximes and amines or nitroso compound due to the rearrangement of oximes. Thus, the FTIR analysis confirms biotransformation of dye into other compounds.

#### Phytotoxicity study

The dye wastewater discharged without treatment into nearby water bodies causes serious environmental problems and health hazards (Mansour et al. 2011). It is clear from Table 2 that up to 100 % germination is being observed in cases of treated wastewater and control (pure water), whereas, only 50 and 10 % germinations were observed for wastewater having dye concentrations 100 and 1,000 mg/l, respectively. Similar observations have been made for plumule and radical for control and dye-contaminated wastewater, whereas treated water shows better growth in plumule as compared to the control. In the case of radical, both control and treated wastewater show nearly similar result. Enhanced growth in plumule in the case of treated water might be due to leftover nutrients after biodegradation.

**Table 2:** Phytoxicity study of Navy N5RL1 dye and its degradation metabolites

	Parameters studies			
	Water	Dye (100 mg/l)	Dye (1,000 mg/l)	Extracted products
Germination	100	55	10	100
Plumule	2.56 ± 0.99	1.2 ± 0.84	0.2 ± 0.17	4.48 ± 0.99
Radical	0.766 ± 0.83	0.23 ± 0.94	0.15 ± 0.08	0.61 ± 0.62

## Conclusion

The isolated bacterial strain *Staphylococcus saprophytic* BHUSS X3 was very efficient in degrading the dye Navy N5RL1 commonly used in carpet industry. The strain is supposed to be capable in degrading other class of the dyes also in the static microaerobic condition. An effluent storage pond near carpet industry without agitation but only amendment of selected locally available bacterial strain could efficiently decrease the dye level.
